# The impact of socioeconomic deprivation on liver transplantation

**DOI:** 10.3389/frtra.2024.1352220

**Published:** 2024-02-27

**Authors:** Paolo De Simone, Giacomo Germani, Quirino Lai, Juri Ducci, Francesco Paolo Russo, Stefano Gitto, Patrizia Burra

**Affiliations:** ^1^ Liver Transplant Program, University of Pisa Medical School Hospital, Pisa, Italy; ^2^ Department of Surgical, Medical, Molecular Pathology and Intensive Care, University of Pisa, Pisa, Italy; ^3^ Multivisceral Transplant Unit, Department of Surgery, Oncology and Gastroenterology, Padua University Hospital, Padua, Italy; ^4^ General Surgery and Organ Transplantation Unit, La Sapienza University of Rome, Rome, Italy; ^5^ Department of Surgery, Gastroenterology, Oncology and Gastroenterology, Padua University Hospital, Padua, Italy; ^6^ Internal Medicine and Liver Unit, Department of Experimental and Clinical Medicine, University Hospital Careggi, University of Florence, Florence, Italy

**Keywords:** liver transplantation, social deprivation, disparities, equity, outcomes

## Abstract

Despite global expansion, social disparities impact all phases of liver transplantation, from patient referral to post-transplant care. In pediatric populations, socioeconomic deprivation is associated with delayed referral, higher waitlist mortality, and reduced access to living donor transplantation. Children from socially deprived communities are twice as much less adherent to immunosuppression and have up to a 32% increased incidence of graft failure. Similarly, adult patients from deprived areas and racial minorities have a higher risk of not initiating the transplant evaluation, lower rates of waitlisting, and a 6% higher risk of not being transplanted. Social deprivation is racially segregated, and Black recipients have an increased risk of post-transplant mortality by up to 21%. The mechanisms linking social deprivation to inferior outcomes are not entirely elucidated, and powered studies are still lacking. We offer a review of the most recent evidence linking social deprivation and post-liver transplant outcomes in pediatric and adult populations, as well as a literature-derived theoretical background model for future research on this topic.

## Introduction

The success of liver transplantation (LT) depends on multiple factors, including medical, surgical, biological, psychological, and social determinants (1). However, the impact of social determinants of healthcare on LT has not been fully explored, though their role in other medical and surgical fields has been largely investigated (2). Understanding the contribution of socioeconomic deprivation (SED) to health outcomes has become an increasingly popular topic of interest (3). Many social and environmental factors affect individual health, including economic stress, limited access to healthcare facilities, poor air quality, high-density housing, inadequate infrastructure maintenance, and lack of safe outdoor spaces (3–6). After controlling for individual medical variables, SED independently predicts specific poor health outcomes. This indicates that social and environmental barriers beyond the control of individual patients play a major role in driving health outcomes (7, 8).

SED is a crucial factor to consider for the best possible outcome of pediatric and adult LT (9). If we can understand the extent to which deprivation characteristics are associated with post-transplant outcomes, we can identify actionable objectives for health improvement (9). This will also help develop fair and equitable metrics for benchmarking and reimbursement and improve the quality of transplant care.

In this review, we use concepts from ethics and public health and examine literature studies to describe the impact of SED on LT outcomes. In addition, we provide a theoretical foundation to direct future studies on this subject.

### Socioeconomic deprivation is a multidimensional construct in constant evolution

SED, or poverty in a broader sense, is the lack of social and economic resources necessary for a good quality of life (10, 11). SED is a complex measure that considers various factors such as individual, family, community, geographical, and national variables, and it depends heavily on the country, ethnicity, and culture (as illustrated in Table 1). SED components are constantly changing, and items are being added according to location (i.e., Europe vs. the US vs. Asia), communities (i.e., neighborhood), culture, study designs, and objectives (12, 13).

**Table 1:** The components of socioeconomic deprivation.

Socioeconomic deprivation
• Socioeconomic status ◦ Low individual and/or household income (adjusted to national/international averages) ◦ Lower individual and/or household education level ◦ Occupied vs. unemployed (if physically active) ◦ Type of occupation
• Housing ◦ Living in a rental property ◦ Crowded housing (usually >4–5 people per 100 square meters)
• Family ◦ Single vs. married/living with a partner ◦ Single parent with minor child(ren)
• Neighborhood ◦ Disadvantaged areas with a lack of community resources limiting or reducing standard quality of life

There are four essential components in SED (11): (1) Socioeconomic status—which includes individual and household income, employment status, and type of occupation; (2) Housing—which refers to living in rental or owned properties and the number of people living together; (3) Family disposition—whether an individual lives alone or with a partner, and the presence of children; and (4) Type of neighborhood—which refers to the availability of community and social resources (i.e., transportation, social services, health facilities, public schools, recreation areas, and activities, etc…). Some authors list additional social factors affecting transplant health outcomes, including racism, discrimination, violence, limited medical knowledge (i.e., medical illiteracy), unstable housing, transportation, childcare, and food insecurity (9).

Although ethnicity does not make up the components of SED, there exists a complex relationship between SED and race because racially segregated communities frequently face discrimination, marginalization, and limited access to healthcare (14). As with non-liver transplantation (15–17), differences in patient and graft survival rates among LT recipients have been well documented in the literature (18, 19). Recently, the US Congress requested that the National Institutes of Health sponsor a study on equity in the US organ transplant system by the National Academies of Sciences, Engineering, and Medicine (20). This was due to significant performance variation, with inequality in race, ethnicity, location, and socioeconomic status (20). Reports on survival after LT have shown differences across race and ethnicity in the US (21). Race has two related meanings in the causal pathways leading to transplant outcomes. Firstly, it is a social classification with observable biological consequences (i.e., graft rejection) that do not always accurately correspond to genetic variation/predisposition (19). Secondly, it is an indicator of potential exposure to systematic racism, implicit bias, and limited access to healthcare (22)^.^ A significant body of literature posits SED to explain outcome differences across racial groups (20). However, the role of socio-economic deprivation (SED) in perpetuating racial disparities in access to and outcomes of organ transplantation remains poorly understood (19).

### Socioeconomic deprivation in the setting of liver transplantation

Research on SED has been conducted in recent years in the US, with investigations in pediatric (23–29) and adult LT populations (30–36). Limited evidence is available from Europe (35), and no information can be derived from Asian countries. In pediatric populations, the impact of SED has been investigated regarding waitlist registrations (25), post-transplant outcomes (23, 26–28), and immunosuppression adherence (29). Additionally, some authors have investigated the effect of environmental risk factors (i.e., air pollution) (24). For adult LT recipients, the focus has been on referral patterns (30, 34), waitlist registrations (31), mode of liver transplant evaluation (33), post-transplant survival (36), quality of life (32) and the risk of post-transplant hepatocellular carcinoma (HCC) recurrence (35).

Table 2 summarizes the findings of the current review.

**Table 2:** The impact of socioeconomic deprivation in pediatric and adult liver transplantation.

Pediatric Populations
Pre-transplantation
• Reduced and delayed referral of children of racial minorities with liver disease (25, 38, 39) • Higher waitlist mortality for children of disadvantaged populations (by 9% per 0.1 increase in deprivation index) (25, 38, 39) • Lower rates of non-standard exception scores for non-White children (38) • Reduced access to living donor liver transplantation for children from racial minorities (25) • Children living >200 miles from transplant centers have a 75% increased risk of waitlist mortality (41).
Post-transplantation
• Children from socially deprived areas are twice as much less adherent to immunosuppressive medication than adherent patients and have up to a 32% increased incidence of graft failure (26, 29). • Transplant centers with a higher proportion of disadvantaged children should implement post-transplant practices to mitigate the impact of socioeconomic deprivation (26). • Living in polluted areas may increase the risk of graft failure or death by up to 54% (24).
Adult Populations
Pre-transplantation
• Reduced rate of referral and waitlisting for socially deprived patients and ethnic minorities (i.e., 8% less per every 0.1 increase in the social vulnerability index) (30). • Reduced transplant rates for socially vulnerable waitlist patients (6% less) (30). • Lack of insurance, unemployment, and social deprivation are associated with a higher risk of not initiating the pre-transplant evaluation, lower rates of waitlisting, and a higher risk of dying without initiating evaluation (33). • Patients from racial minorities have a 31% higher risk of not being listed for transplant than White individuals (33). • Alcohol-related liver disease patients from deprived areas are less likely to be listed for transplantation and have an increased rate of waitlist mortality (31).
Post-transplantation
• Social deprivation is linked to lower survival rates within 2 years after transplantation (43), reduced quality of life (32), and higher rates of anxiety and depression (32). • Overall evidence linking SED to post-transplant outcomes is numerically limited and weaker than for pediatric populations (30–36, 43). • The evidence that shows a connection between racial disparities and post-transplant outcomes is more compelling (45–48). Black recipients have an increased risk of post-transplant mortality by up to 21% (48).

## Pediatric populations

### Access to pre-transplant care, wait-listing registrations and pre-transplant mortality

Based on literature data, SED accounts for limited or delayed access to pre-transplant care, and the impact of neighborhood deprivation is particularly significant for racial/ethnic minority children (25). Black and Hispanic children have higher lab PELD/MELD scores than White children, indicating possible delays in referral, listing, or transplantation (9, 25, 28). In a recent, extensive registry analysis of 7,716 patients, Black and Hispanic children had increased unadjusted hazard of waitlist mortality than White children [subhazard ratio (sHR) = 1.44; 95% CI = 1.18–1.75 for Black patients and sHR = 1.48; 95% CI = 1.25–1.76 for Hispanic children, respectively] (25). However, after adjusting for neighborhood deprivation, insurance, and Model for End-Stage Liver Disease (MELD)/Pediatric End-Stage Liver Disease (PELD), Black and Hispanic children did not have increased hazard of waitlist mortality (sHR, 1.12; 95% CI, 0.91, 1.39 and sHR, 1.21; 95% CI, 1.00, 1.47, respectively) (25). Similarly, Black and Hispanic children had decreased likelihood of LDLT (sHR, 0.58; 95% CI, 0.45, 0.75 and sHR, 0.61; 95% CI, 0.49, 0.75, respectively), but adjustment attenuated the effect of Black and Hispanic race/ethnicity on likelihood of LDLT (sHR, 0.79; 95% CI, 0.60, 1.02 and sHR, 0.89; 95% CI, 0.70, 1.11, respectively) (25). These results indicate that incorporating SED and disease severity into the model reduces the differences observed between children of different ethnicities (25). However, a more nuanced understanding of how neighborhood adversity impacts clinical results is still lacking (25).

Non-standard exception requests may also explain waitlist disparities (9). In 2019, 75% of pediatric transplant recipients had an exception score at the time of transplant (37). Still, children of non-White race, including Black, Hispanic, Asian, American Indian/Alaska Native, Native Hawaiian/Other Pacific Islander, and multiracial children, had 13% lower rates of exception score requests submitted by the transplant team (38). Children with exception approval have a decreased risk of graft loss, while those with exception denial have a higher risk of post-transplant death (39).

Another potential contributor to waitlist disparities may be differential access to living donor liver transplantation (LDLT). For Black children, Mogul et al. found a reduced incidence of LDLT vs. White children utilizing the Scientific Registry of Transplant Recipients (SRTR) (40). This was confirmed by Wadhwani et al., who reported that Black and Hispanic children were about half as likely to undergo LDLT compared to White children (sHR = 0.58; 95% CI = 0.45–0.75 for Black children and sHR = 0.61; 95% CI = 0.49–0.75 for Hispanic recipients, respectively) (28). This disparity in LDLT rates for children may lead to longer wait times, higher waitlist morbidity and mortality, and inferior post-transplant graft and patient survival (9).

Neighborhood SED has also been associated with adverse outcomes before transplantation. In unadjusted analyses, each 0.1 increase in the deprivation index was associated with a 9% increased sub-hazard of waitlist mortality (28). The distance to care also impacts waitlist mortality. Children over 200 miles from their transplant center have a 75% higher risk of waitlist death, possibly reflecting a delay in referral or listing for transplantation (41). Notably, in their recent paper, Henson et al. highlighted several potential barriers to evaluating and selecting for LT, including poverty, educational attainment, access to healthy food, and access to technology (34).

### Post-transplant care and outcomes

Social adversity has a negative impact on the outcomes of LT. Using SRTR data, Wadhwani et al. found that neighborhood SED was associated with an increased risk of graft failure and death over a 10-year timespan after transplant (28). In multivariable analysis adjusted for race, each 0.1 increase in the deprivation index was associated with a 11.5% (95% CI: 1.6%–23.9%) increased hazard of graft failure and a 9.6% (95% CI: −0.04% to 20.7%) increased hazard of death. Notably, when the proportion of patients from SED neighborhoods increases for a given transplant center, patients have a 32% increased hazard of graft failure (26). However, the impact of neighborhood disadvantage can be mitigated by medical practices. High-performing pediatric liver transplant centers achieved good long-term outcomes despite caring for socioeconomically deprived children, demonstrating that there may be transplant center practices that mitigate the risks of SED (26). Addressing treatment non-adherence may be one such strategies, since pediatric recipients living in the most deprived neighborhood deprivation index quartile were twice as likely to be non-adherent to immunosuppressive medication (29). The insurance status has an additional impact on post-transplant outcome, and children less than 18 years old with Medicaid insurance have a relative risk of 1.42 of post-transplant mortality (42).

Living in neighborhoods with high air pollution can increase the risk of graft failure and death post-transplant in children, even after accounting for sociodemographic factors (24). Recently, Yalung et al. investigated the role of environmental factors and found that air pollution was linked to a 54% higher risk of graft failure or death (HR: 1.54; 95% CI: 1.29, 1.83; *P* < 0.001) after adjusting for race, insurance status, rurality, and neighborhood socioeconomic status (24).

## Adult populations

### Access to pre-transplant care, wait-listing registrations and pre-transplant mortality

The socioeconomic status of individuals and communities significantly impacts the outcomes of the pre-transplant evaluations (30). For every 0.1 increase in the overall Social Vulnerability Index (SVI), there was an 8% reduction in the rate of waitlisting, as per HR 0.92 (95% CI 0.87–0.96, *p* < 0.001) (30). The domains significantly contributing to this correlation were socioeconomic status, household characteristics, housing type, transportation, and racial and ethnic minority status (30). Patients from vulnerable communities had a 6% lower transplant rate (HR 0.94, 95% CI 0.91–0.98, *P* = 0.007), and socioeconomic and household characteristics in the SVI domain significantly contributed to this association (30). At the individual level, lack of government insurance and unemployment were associated with lower rates of waitlisting and transplantation, but there was no association with mortality before or while on the waitlist (30).

According to a recent analysis carried out at a single center, liver disease patients who live in socially deprived communities have a higher risk of not being listed compared to patients with higher socioeconomic status (33). The analysis found that patients from socially deprived neighborhoods are also at a greater risk of not initiating the evaluation post-referral and dying without initiating the evaluation (33). The results showed that the adjusted relative risk (aRR) for not being listed was 1.14 (95% CI = 1.05–1.22, *P* < 0.001). The aRR for not initiating the evaluation post-referral was 1.20 (95% CI = 1.01–1.42, *P* = 0.03). Lastly, the aRR for dying without initiating evaluation was 1.55 (95% CI = 1.09–2.2, *P* = 0.01) (33). The study found that White patients with low SED have similar rates of being listed compared to White patients with high SED. However, patients from social minority groups who live in neighborhoods with low SED are 31% more likely not to be listed for a transplant compared to patients from the same minority group living in neighborhoods with high SED. The results were statistically significant (aRR = 1.31; 95% CI = 1.12–1.5; *P* < 0.001) (33).

The impact of social adversity appears to be greater for certain indications to LT. In a recent analysis of the UNOS registry 2008–2019, Cullaro et al. have shown that patients from the most deprived areas are the least likely to be listed for alcohol-related liver disease (OR = 0.97, 95% CI = 0.95–0.98) and have an increased rate of waitlist mortality (OR = 1.1; 95% CI = 1.06–1.14) (31).

### Post-transplant care and outcomes

Compared to pediatric patients, there is limited information on how social adversity impacts adult LT recipients. Initial surveys (1987–2001) on the influence of neighborhood income, education, and insurance showed that education had a marginal influence on outcomes, and patients with Medicare and Medicaid had lower survival than those with private insurance (36). More recently, in a proportional hazards model analysis, LT recipients with the lowest socioeconomic status have an increased risk of death within 2 years after transplantation (HR = 1.17; 95% CI = 1.02–1.35) (43). After adjusting for differences in recipient characteristics, donor organ quality, transplant center volume/quality, geographic region, and DSA, being in the lowest SED quartile remained an independent predictor for patient but not graft survival (43). Adjusting for individual hospitals had minimal impact on patient survival hazard ratio, indicating that differences in SED groups did not result from hospital care (43).

Health-related quality of life (HRQoL) seems poorer in recipients from disadvantaged areas. In a recent publication, Sgrò et al. investigated HRQoL in 331 patients and found that greater SED was associated with lower post-transplantation HRQoL scores, with a difference of 9.7 points (95% CI: 4.6–14.9, *P* < 0.001) between the most and least deprived quintiles (32). Recipients living in areas of least deprivation were less likely to suffer from anxiety (OR = 0.05; 95% CI: 0.00–0.28; *P* = 0.003) or depression (OR = 0.13, 95% CI = 0.02–0.56; *P* = 0.009) (32).

While socially disadvantaged patients with HCC show inferior survivals (44), a group of French investigators failed to show any impact of SED on the post-transplant outcome of patients with HCC (35). In their registry analysis of 3,865 recipients, the European deprivation index (EDI) did not impact overall survival after LT for HCC, while the number of tumor nodules and time on the waiting list were the independent prognostic factors predicting survival (35).

The literature has extensively analyzed the impact of race and ethnicity, particularly in the US context. Black patients have worse outcomes than White patients, including lower graft function (45), inferior graft survival (46), and worse overall survival (47), revealing the role of racial disparities. This disparity has remained consistent over time (i.e., before and after the MELD era) (47) and persists after controlling for patient-level factors, such as socioeconomic status (43) and clinical covariates (45). Recent reports confirm that Black patients have a 21% higher mortality risk than White patients, but no effect modification by transplant center volume was found (48).

## A theoretical background model to explain the impact of socioeconomic deprivation

Although evidence shows socioeconomic determinants impact LT outcomes, their mechanisms in clinical practice remain elusive. However, a clear understanding by clinicians is crucial for equitable patient outcomes in liver disease and transplantation. To ensure equitable care and outcomes in LT, having a common language across the transplant community is crucial. Understanding the social determinants of health and engaging in integrated person-centered liver patient care across classical medical specialty boundaries is essential, which can help identify pre-transplant patients and recipients requiring more intensive resources (49). This will help equalize outcomes and ensure everyone receives the care they need. Still, reappraisal of disease-related medical challenges must be conducted to increase awareness among liver transplant physicians and reduce the social stigmatization associated with liver disease (49, 50).

**Figure 1:**
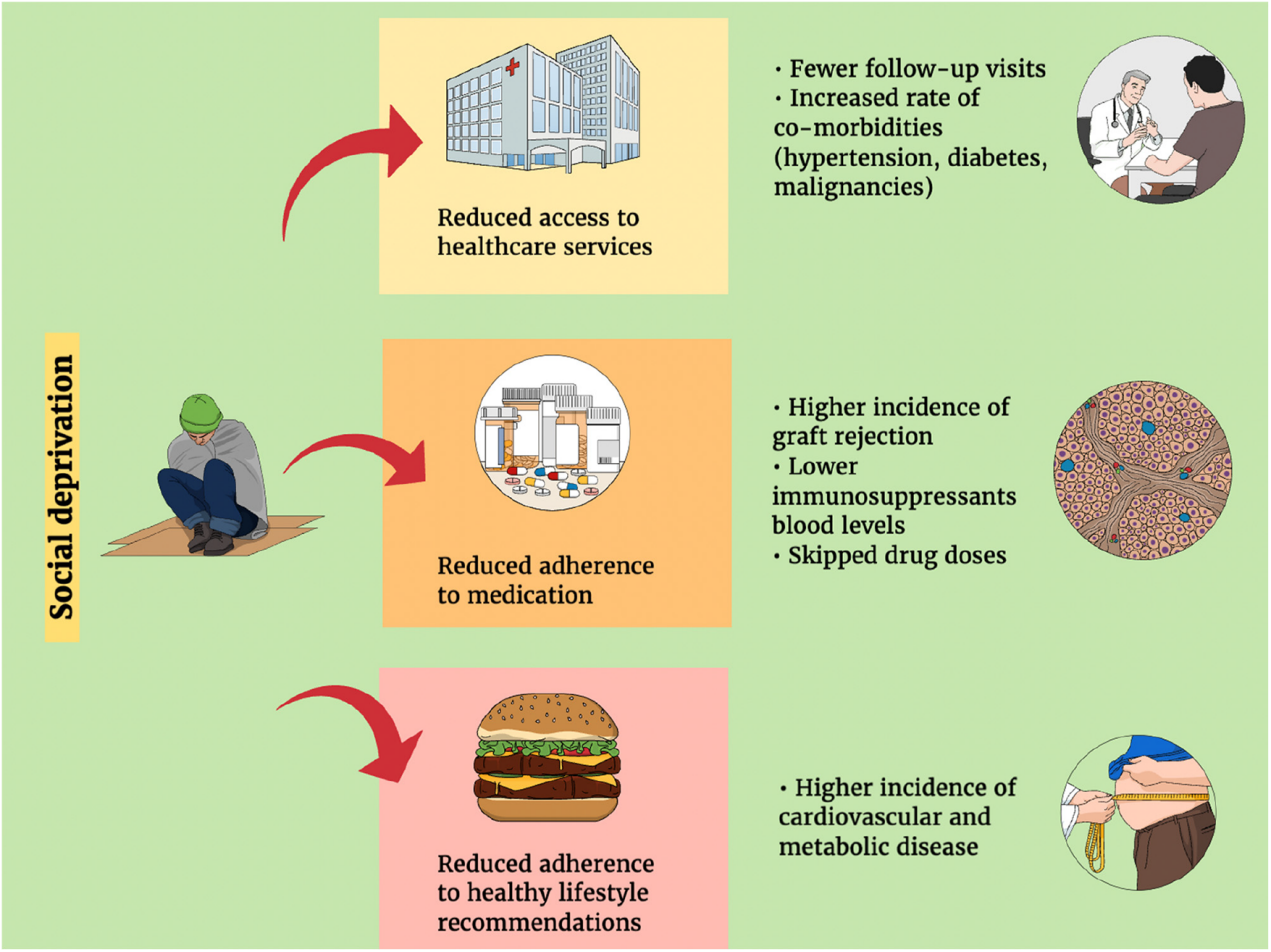
The implications of socio (economic) deprivation on the outcomes of liver transplantation.

Our literature search identified three areas in which SED may affect the results of LT. These areas ought to be considered as potential topics for future research and interventions (Figure 1).

### Access to health care services

In transplant (9, 16, 25, 30, 31, 33, 34, 36) and non-transplant populations (51, 52–54), SED is associated with reduced access to healthcare services and facilities. Liver disease patients from deprived areas and minorities are waitlisted in greater severity per PELD/MELD scores (9, 25, 30, 39, 40) and less frequently referred to living donor LT (25). Social barriers, such as stigma (i.e., negative or discriminatory attitudes of others), have a significant impact on liver diseases and patients' referral patterns, leading to discrimination, reduction in health-care-seeking behavior, and reduced allocation of resources, which all result in poor clinical outcomes (49). Due to delayed referral to appropriate care, waitlist mortality is higher in socially deprived populations (25, 31, 33, 39, 40). It has also been noted that a greater distance from the transplant center where a patient was referred to may worsen their disease severity (41).

Some indications of liver transplantation still bear the consequence of public stigma and SED. This is particularly true for alcohol use disorders, acute alcohol-related hepatitis, alcohol-related chronic liver disease, and metabolic dysfunction-associated steatotic liver disease (MASLD) (49, 50, 55, 56). Globally, there has been a significant increase in alcohol use disorder among women, ethnic and racial minorities, and individuals living in poverty, who also experience poor access to alcohol treatment (56). This has resulted in a rise in alcohol-related liver diseases (56). Rising rates of MASLD and associated fibrosis have been observed in Hispanics, women aged >50, and individuals experiencing food insecurity (56). Limited access to viral hepatitis screening and treatment for racial and ethnic minorities, as well as uninsured or underinsured individuals, leads to higher mortality rates and later diagnoses of HCC (56). However, favorable results can be obtained after LT through an accurate selection of transplant candidates, multidisciplinary integration of pre-transplant care with family support, and close post-transplant integrated care (57). People with alcohol-related disorders are subject to public stigma, self-stigma (negative attitudes, including shame, about their condition), and structural stigma (policies that intentionally or unintentionally limit opportunities for people with the disease) (58). Patients with alcohol-related liver disease often describe healthcare settings as stigmatizing, and removing blaming for alcohol use is central to facilitating access to healthcare and transplantation (49, 50, 58). Furthermore, a significant proportion of individuals with alcohol-related disorders are from historically underrepresented racial/ethnic, sex/gender, and sociocultural groups and those vulnerable in their social determinants of health, creating additional barriers to treatment (50).

In the post-transplant period, deprivation and racial disparities are associated with reduced graft and patient survival, especially for pediatric populations (25, 26, 29). Increased non-adherence to immunosuppression (29, 59) and the impact of environmental disadvantages (24) could be contributing factors, but additional mechanisms should be considered. Quality and quantity of care within the household (60), availability of caregivers and resources (60), attending follow-up visits (61), and involvement of other health care practitioners (61), such as recipient coordinators, pharmacists, dermatologists, and addiction specialists (61), may improve outcomes. Peer and social pressure on pediatric/adolescent recipients (62) and cognitive determinants of health status and adherence (63) are interesting areas that require further investigation. Higher SED may disincentive adherence to pre- and post-transplant lifestyle recommendations, leading to worse outcomes due to the role of non-surgical and non-medication risk factors on liver disease and transplant outcomes (64). In the population of patients with alcohol use disorders, the available literature has documented a higher relapse rate post-transplantation for patients with lower social support and socioeconomic status through a complex interplay of social, economic, psychological, and behavioral mechanisms (65). Post-transplant recurrence of MASLD has been associated with metabolic derangements such as insulin-dependent diabetes, hyperlipoproteinemia, and graft steatosis within 2 years after transplantation (66). The role of social deprivation in this setting still needs clarification.

### Immunosuppression non-adherence

Extensive literature evidence in transplantation shows that higher SED (29, 58, 67, 68), being divorced, having a history of substance or alcohol use, having mental health needs, missing clinic appointments, and not maintaining medication logs are associated with a higher probability of immunosuppressive medication non-adherence (69).

Health literacy refers to an individual's ability to access, understand, and use health-related information and services to make informed decisions about their health. This has recently been recognized as a critical factor in treatment compliance and overall health-related quality of life (70). Inadequate adherence to treatment due to difficulties in understanding medical information related to medication and lifestyle recommendations following organ transplantation or the inability to find appropriate information can increase the risk of re-hospitalization and transplant organ failure (71). Health literacy follows a socioeconomic gradient (72), and it might be speculated that transplant individuals from socially deprived areas are more exposed to illiteracy and consequent increased treatment non-adherence. To date, this hypothesis must be investigated in LT recipients.

Decreased adherence after kidney transplantation is associated with black race (73), but also with transplant center and dosing frequency (73). Investigating non-adherence to immunosuppression after transplantation is challenging, requiring direct measurements (such as electronic monitoring), blood drug exposure level testing, and collateral reports (such as patients' self-reports and/or caregivers' opinions) (59). Longitudinal observations should be conducted and correlated with the socioeconomic determinants of interest in each transplant population. In the case of a liver transplant, detecting the impact of non-adherence might be more challenging due to the immunologic privilege of the liver graft and the lower probability of acute cellular rejection compared to other solid organ transplant categories (74).

### Non-adherence to lifestyle recommendations

Reduced adherence to lifestyle recommendations can be linked to social deprivation, a third area that needs to be explored. Along with constant medical care, the active participation of patients in their healthcare plan and adherence to lifestyle recommendations are crucial for improving the outcomes of LT (75). To reduce the rate of chronic attrition after a transplant, it is important to address pre-existing health conditions and complications that may arise from immunosuppressive drugs (76). This involves managing conditions like diabetes, hypertension, and obesity and making healthy lifestyle choices, such as quitting smoking, engaging in regular physical activity, and following a healthy diet. It is also crucial to seek prompt medical attention from the transplant center. From previous research in non-transplant populations, children living in socially deprived areas have a twofold higher risk of obesity than children from families with higher socioeconomic status (77). Likewise, children from families with medium and low education have twice the risk for obesity compared to children with high parental education (77). In the UK, areas with high rates of obesity are often concentrated around economically depressed urban areas in the north of England, leading to health inequalities across the country (78). Therefore, we may speculate that SED act along the same trajectories in transplant populations by contributing to an increase in the negative impact of co-morbidities and immunosuppression and non-immunosuppressive medication complications.

### Future directions

Addressing the negative consequences of social adversity on the outcomes of LT requires comprehensive strategies that are multi-level and multi-dimensional. These strategies should be based on the chronic care model (CCM) and should cover the entire continuum of LT care, starting from the pre-transplant phase and extending to the long-term follow-up.

All levels, including patients, caregivers, stakeholders, clinics, academia, communities, and institutions, should be involved, and interventions should address the biological, psychological, and social determinants of transplant outcomes. To a greater extent, the US National Academies of Sciences, Engineering, and Medicine have recently laid out the details of these initiatives, addressing all strata of patients (79).

(1) Increase awareness. The first strategy is to raise healthcare professionals' awareness of SED's impact on LT outcomes. This requires promoting research, data measurement, and exchange among clinicians/researchers. Standardizing research language and measures across institutions and countries is crucial due to SED components' varied definitions and implications. Research on the interaction between the environment (i.e., air/water pollution, food insecurity) behaviors (i.e., diet, exercise) and biology (i.e., immune response, post-transplant cancer risk) should be promoted by academia, scientific societies, and research institutions.
(2) Adjust care delivery to patients' needs. The second initiative aims to provide sustainable, patient-centered care that minimizes routine disruption to patients and their families (i.e., care adjustment). Socioeconomic profiling should be integrated into pre- and post-transplant care via tailored questionnaires or census block deprivation indexes. Adjusting care to patients' needs also requires removing obstacles hindering communication/interaction between patients and transplant centers. For instance, telehealth appointments can be used instead of in-person visits, tailored to the patient's health status and distance from the transplant center. Transplant centers should also establish collaborative networks with local referring institutions to increase adherence to follow-up visits for patients from socially disadvantaged areas. They should also improve data exchange and promote crosstalk among multiple institutions. Engaging family caregivers from the beginning of the transplant process is crucial to ensuring patient participation in pre- and post-transplant care.
(3) Advocacy. The transplant community should advocate for eliminating inequities to access to pre and post-transplant care by introducing norms/regulations, social (i.e., housing and transport vouchers), and financial (i.e., reimbursements) incentives for patients from socially disadvantaged areas and seeking transplant care. We also recommend that deprivation indexes (both individual and at the census block levels) be introduced in the case-mix evaluation to better understand the connection between socioeconomic disadvantage (SED) and transplant outcomes. Public, personal, and structural stigma should also be removed to enhance access to pre- and post-transplant care for patients with alcohol-related liver disease and disorders and for those with social disadvantages. Public discourse on the role of social determinants in transplant care, both nationally and internationally, should be favored among clinicians, patients, caregivers, and stakeholders.

## Conclusions

Personalizing LT recipient care based on medical, surgical, immunologic, psychologic, and socioeconomic factors improves outcomes. There is growing evidence, derived mainly from studies in the US, that socioeconomic deprivation plays a crucial role in delaying prompt patient referral, hindering access to care, and discouraging participation in follow-up care. Public, personal, and structural stigma should also be removed to enhance access to pre- and post-transplant care. The socioeconomic profile of liver disease patients seeking transplant care should be integrated into the pre-transplant evaluation process and their post-transplant care plan.
